# Thermophilic-mesophilic temperature phase anaerobic co-digestion compared with single phase co-digestion of sewage sludge and food waste

**DOI:** 10.1038/s41598-024-62998-w

**Published:** 2024-05-25

**Authors:** Yangqing Hu, Ce Shen

**Affiliations:** 1 Fair Friend Institute of Intelligent Manufacturing, Hangzhou Vocational and Technical College, Hangzhou, 310018 China; 2 Hangzhou Huaxin Mechanical and Electrical Engineering Co., Ltd, Hangzhou, 310030 China; 3 College of Electronic Information and Optical Engineering, Nankai University, Tianjin, 300350 China

**Keywords:** Environmental sciences, Energy science and technology

## Abstract

Anaerobic co-digestion is an effective method for addressing the issue of a single substrate not being able to achieve optimal conditions for anaerobic digestion. By adjusting the mixture ratio of sewage sludge and food waste to achieve the optimal carbon to nitrogen ratio, the effectiveness of thermophilic–mesophilic temperature phase anaerobic co-digestion (TPAcD) was evaluated in comparison to single phase mesophilic anaerobic co-digestion (MAcD) and thermophilic anaerobic co-digestion (TAcD). The results indicated that TPAcD increased methane yield by 50.3% and 32.7% compared to MAcD and TAcD, respectively. The variation in VFA, pH, and ammonia nitrogen levels demonstrated that TPAcD combines the advantages of both MAcD and TAcD, with a higher hydrolysis rate in the early stage under thermophilic conditions (55 °C) and a suitable environment in the later stage under mesophilic conditions (35 °C). The kinetic parameters of anaerobic co-digestions also demonstrated that TPAcD performs better. Therefore, further research on TPAcD of sewage sludge and food waste is warranted due to its significant improvements in methane production rate, total methane yield, and system stability. Additionally, TPAcD contributes to reducing carbon emissions and supports the realization of “carbon neutrality”.

## Introduction

With the rapid development of society and the exponential growth of the population, the amount of solid waste has also increased significantly, making it a global issue^1^. According to reports, it is expected that 3.4 billion tons of solid waste will be generated annually by 2050, with organic waste accounting for the majority at around 46%^2^. Sewage sludge and food waste are two common types of organic waste. Based on the statistical investigations, highly populated countries generate over 60 million tons of sewage sludge^3^. Meanwhile, approximately one third of total food production (1.3 billion tons) is wasted, resulting in an economic loss of over 750 billion dollars annually^4^. In recent decades, a considerable amount of research has focused on the resource utilization of organic waste to recover energy, resulting in the proposal of various disposal methods such as incineration, composting, and anaerobic digestion (AD)^5^. Among these methods, AD of organic waste has been widely implemented to recover energy in the form of biogas and reduce negative environmental impacts^6^.

AD is a complex biochemical process that occurs in the absence of oxygen and involves four stages: hydrolysis, acidogenesis, acetogenesis, and methanogenesis^7^. One of the main factors that affects the efficiency of AD is temperature. Most industrial application are conducted as mesophilic (35–37 °C) AD (MAD), and thermophilic (50–55 °C) AD (TAD) attracts growing attentions. TAD has been found to have higher biomethane production, a faster reaction rate, higher organic reduction, and a shorter retention time compared to MAD. However, TAD also requires more energy to heat the digester and can be more unstable due to the accumulation of volatile fatty acids (VFA) and inhibition by free ammonia^8,9^. To address these issues, a two-phase AD process called thermophilic–mesophilic temperature phase AD (TPAD) has been developed. TPAD consists of a short thermophilic phase followed by a longer mesophilic phase^10^. The thermophilic phase speeds up the rate-limiting hydrolysis stage, while the mesophilic phase provides stable conditions for the subsequent stages^11^. Previous research has shown that TPAD has positive effects on biogas production, organic reduction, and system stability^12,13^.

The carbon to nitrogen ratio (C/N) is a crucial factor that affects the efficiency of anaerobic digestion (AD). A higher C/N ratio can lead to acid inhibition, while a lower C/N ratio can cause ammonia inhibition. Many studies have identified the optimal C/N ratio, which ranges from 10 to 35^14–16^. However, a single substrate typically has a lower or higher C/N ratio, which can be improved by combining two or more substrates with complementary properties. This can result in a “1 + 1 > 2” synergistic effect. Therefore, anaerobic co-digestion (AcD) is commonly used to balance the C/N ratio. Sewage sludge and food waste are both representative organic wastes, with sewage sludge having a lower C/N ratio and food waste having a higher C/N ratio^16^. Thus, co-digestion of these two wastes can lead to better AD performance. While numerous scholars have studied the AcD of sewage sludge and food waste, most of the research has been conducted under single-phase mesophilic or thermophilic conditions. Since TPAD has better performance than MAD or TAD^17^, it’s necessary to study the thermophilic–mesophilic temperature phase anaerobic co-digestion (TPAcD) of sewage sludge and food waste to further improve the energy recovery efficiency.

Several studies have been conducted on TPAcD. For example, Tena et al.^18^ compared the TPAcD of sewage sludge and wine vinasse with single-stage AcD within the framework of the circular economy; Borowski^19^ studied the TPAcD of the hydromechanically separated organic fraction of municipal solid waste with sewage sludge, focusing on the influence of retention time on the thermophilic stage; Sillero et al.^20^ researched the TPAcD of sewage sludge, wine vinasse, and poultry manure, with a detailed discussion on the effect of retention time on the mesophilic stage. However, there is still a lack of information on TPAcD of sewage sludge and food waste, and further research is needed to improve our understanding in this area.

Thus, the main objective of this study is to compare the performance of TPAcD of sewage sludge and food waste with single phase mesophilic anaerobic co-digestion (MAcD) and thermophilic anaerobic co-digestion (TAcD). The methane production, VFA variation, pH fluctuation, and ammonia nitrogen variation were experimented to evaluate the performance of AcD. Moreover, the kinetic modeling was used to estimate the kinetic parameters of AcD. The electricity generation and avoided GHG emissions were calculated to determine the potential for carbon emission reduction through AcD. The results of this study aim to characterize the TPAcD process of sewage sludge and food waste and further improve the resource utilization efficiency of the two typical wastes, leading to the achievement of “carbon neutrality”.

## Materials and methods

### Materials

The sewage sludge was derived from a sewage treatment plant in Hangzhou, China. The sewage sludge was characterized by a total solid (TS) of 17.82%, a volatile solid (VS) of 8.37%, a pH of 7.08 and a C/N of 6.56. The food waste was collected from a school kitchen in Hangzhou, China. After removing inorganic matters, the food waste was crushed using a food agitator. The food waste was characterized by a TS of 23.54%, a VS of 21.85%, a pH of 5.74 and a C/N of 21.46. To ensure consistency, all samples were stored at 4 °C before further testing.

### Experimental set-up

**Figure 1:**
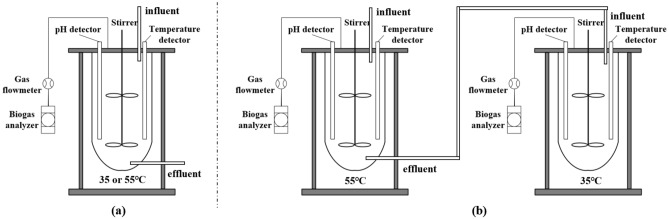
(**a**) Schematic of the single-phase anaerobic digestor; (**b**) schematic of the thermophilic–mesophilic temperature phase anaerobic digestor.

The effects of AcD were examined using five 5 L anaerobic digestors. 2 L of mixture and 1 L of inoculated sludge were added to each anaerobic digestor. The TS content of the mixture was adjusted by adding pure water. The initial pH was adjusted to 7.0 by adding either NaOH or HCl. Figure 1a shows the schematic of the single-phase anaerobic digestor. The temperature was controlled by an external circulation heating system at either 35 °C (mesophilic) or 55 °C (thermophilic). A 50-rpm stirring device was used to ensure proper mixing in the digestors. Biogas composition and production were measured daily. Figure 1b shows the schematic of the thermophilic–mesophilic temperature phase anaerobic digestor. Two single-phase anaerobic digestors were connected. The thermophilic (55 °C) digestor was operated at 2 days of retention time, its effluent was used to feed for the following mesophilic (35 °C) digestor. To measure VFA and ammonia nitrogen, samples were collected from the digestors at regular intervals. To ensure the repeatability of the results, three replicates of each sample were tested and the average values were calculated.

### Experimental process

Initially, the optimal C/N ratio of AcD was studied. As shown in Table 1, sewage sludge and food waste were mixed in six different ratios and then subjected to single-phase mesophilic and thermophilic anaerobic digestion. Based on the methane production, the optimal mixture ratio was selected for use in the subsequent experiment. Three groups of mixtures were then tested: MAcD, TAcD, and TPAcD. The performance was evaluated by measuring methane production and monitoring changes in VFA, pH, and ammonia nitrogen levels.

**Table 1:** Different mixture ratio of sewage sludge and food waste.

No	Mixture ratio of sewage sludge and food waste (by TS)	C/N
1	100:0	6.56
2	80:20	9.54
3	60:40	12.52
4	40:60	15.50
5	20:80	18.48
6	0:100	21.46

### Analysis

TS and VS were determined according to the American Public Health Association (APHA) standard^21^. The elemental analysis of sewage sludge and food waste was conducted using an elemental analyzer (Thermo NA 2100, USA). The composition of the biogas was identified by a biogas analyzer (Geotech Biogas 5000, UK). The ammonia nitrogen was measured by a multi parameter water quality comprehensive measuring instrument (LOVIBOND ET99731, German). The VFA was analyzed by a gas chromatograph (Shimadzu GC 2014C, Japan) with flame ionization detector^22^.

### Kinetic modeling

The Gompertz model was widely applied to estimate the kinetic parameters of anaerobic digestion^23^.

$$\frac{V}{{V}_{m}}={e}^{-{e}^{\frac{S\cdot e}{{V}_{m}}\left(D-t\right)+1}}$$

where V represents the methane yield at time t, mL g^−1^ VS; V_m_ is the maximum methane production potential, mL g^−1^ VS; t stands for the operation time, d; S is the maximum methane production rate, mL g^−1^ d^−1^; D is the lag time, d; e is Euler’s constant.

The correlation coefficient R^2^ was calculated to evaluate the fitting performance of the kinetic modeling, as follows:

$${R}^{2}=\frac{\sum {({\widehat{y}}_{i}-\overline{y })}^{2}}{\sum {({y}_{i}-\overline{y })}^{2}}$$

### Electricity generation and avoided GHG emissions

According to the experimental data obtained from this research, the potential of electricity generation (EG) from burning methane was estimated. And the mitigation of greenhouse gases (GHG) related to the replacement of the power obtained from the grid by methane was evaluated^18^.

The potential of electricity generation (EG) was calculated as following:

$$EG={Q}_{methane}\times {LCV}_{methane}\times {n}_{g}\times {n}_{e}/CkW$$

where EG is the electricity generation, kWh/t; Q_methane_ is the volume of methane, m^3^; LCV_methane_ is the lower calorific value of methane (8500 kcal/m^3^); n_e_ is the engine efficiency (45%); n_g_ is the generator efficiency (95%); and CkW is the conversion factor from kcal to kWh (860 kcal/kWh).

The avoided GHG emissions (AGHG) parameter indicates the potential reduction in greenhouse gas emissions by the production plant considering the use of biogas from biomass conversion instead of non-renewable energy sources. The avoided GHG emissions due to electricity generation were obtained as following:

$${A}_{GHG}=0.2768\times EG$$

where 0.2768 is the emission factor in kg CO_2_eq/kWh attributed to the favorable impact of the electricity generated by methane^18^; EG is the electricity generation, kWh/t.

## Results and discussion

### Influence of C/N on mesophilic and thermophilic anaerobic co-digestion

**Figure 2:**
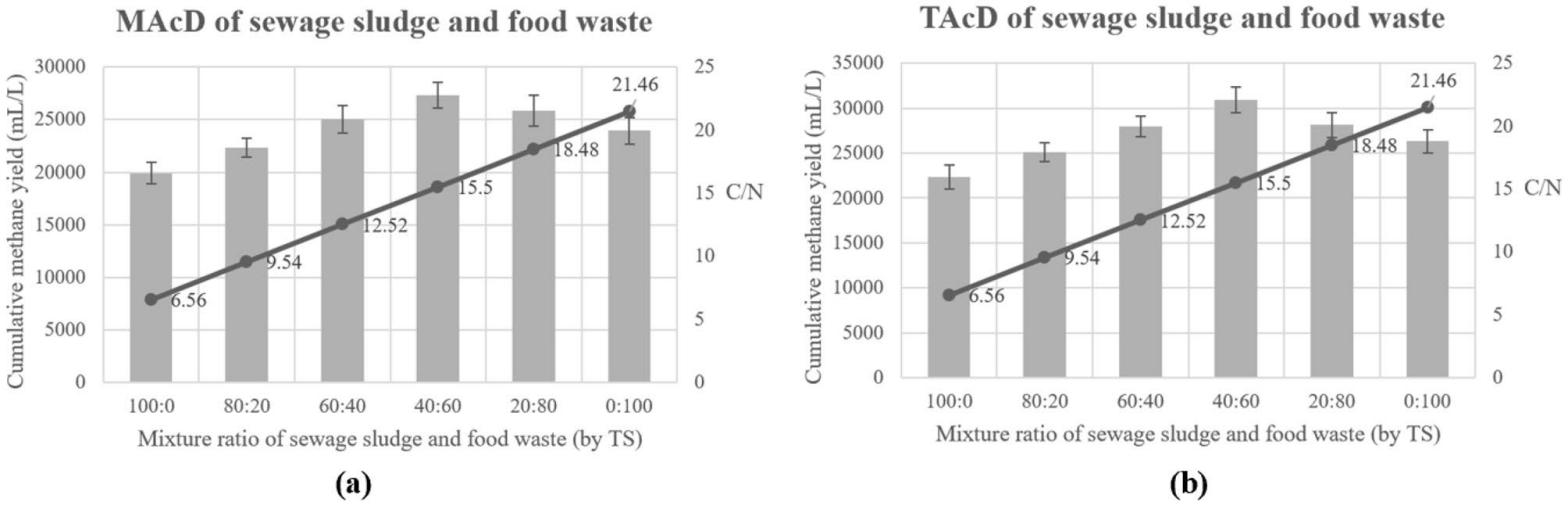
(**a**) Influence of C/N on MAcD; (**b**) influence of C/N on TAcD.

Six different groups were tested using varying ratios of sewage sludge and food waste under both mesophilic and thermophilic conditions to determine the optimal C/N ratio. As shown in Fig. 2, the optimal mixture ratio for both MAcD and TAcD was found to be 40:60, resulting in an optimal C/N ratio of 15.5. This optimal group showed an increase in methane yield of 14.1–38.1% compared to single substrate groups. These findings were consistent with previous reports^24,25^, and highlight the importance of achieving the optimal C/N ratio when mixing sewage sludge and food waste for stable operation of the AD system. Maintaining an appropriate C/N ratio is crucial for promoting efficient metabolism of AD microorganisms, enhancing the activity of biological enzymes, and effectively breaking down complex organic compounds such as carbohydrates, proteins, and oils. This ultimately leads to improved biogas production^26^. The optimal mixture ratio of sewage sludge and food waste was then applied in subsequent experiments.

### Methane yield of anaerobic co-digestion

**Figure 3:**
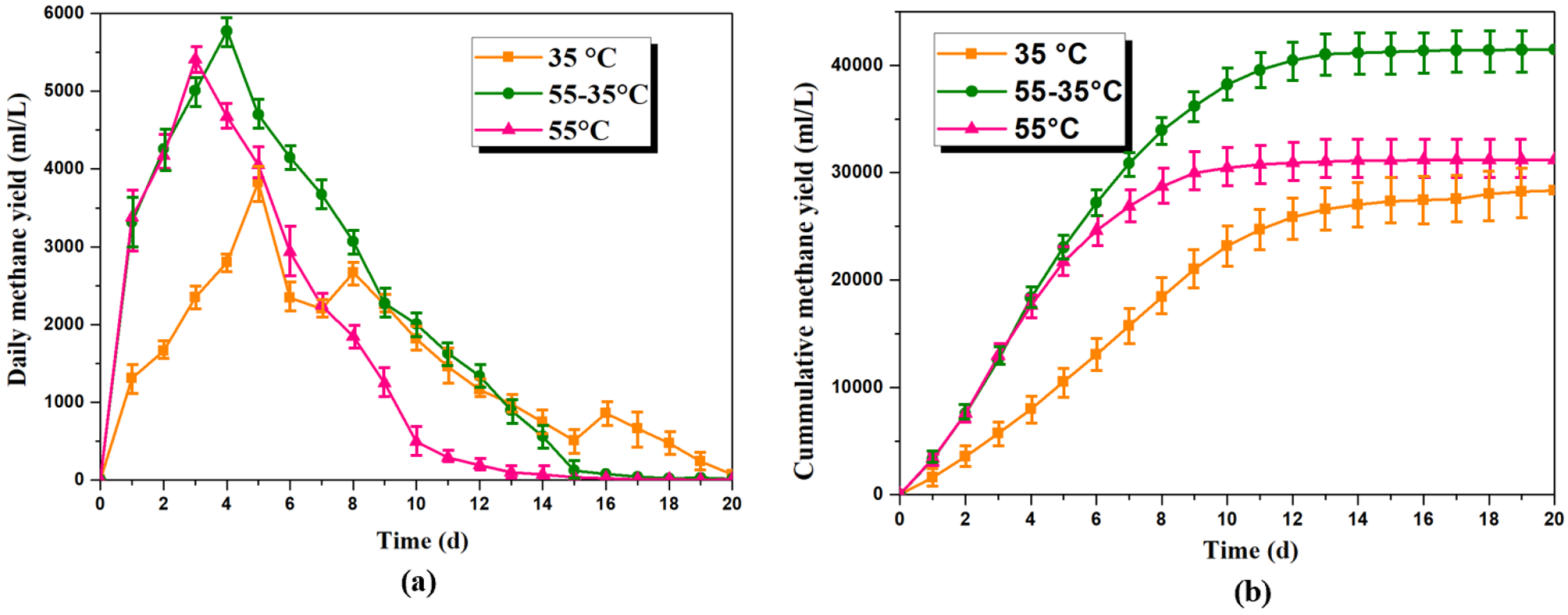
(**a**) Daily methane yield of MAcD, TAcD and TPAcD; (**b**) cumulative methane yield of MAcD, TAcD and TPAcD.

After obtaining the optimal ratio, the chosen mixture of sewage sludge and food waste (40:60, C/N = 15.5) was used in the TPAcD process. Figure 3 shows the daily and cumulative methane yield of MAcD, TAcD, and TPAcD. The curves indicate that TAcD (55 °C) had a rapid initial methane production rate, reaching its peak on the 3rd day. However, there was a rapid decrease in methane production after the 11th day. MAcD (35 °C) was relatively stable compared to TAcD, with a lower initial methane production rate and a longer reaction period. TPAcD (55–35 °C) combined the advantages of both MAcD and TAcD, with a rapid initial methane production rate under thermophilic conditions (55 °C) in the first two days, and a stable process under mesophilic conditions (35 °C). As a result, the cumulative methane yield of TPAcD was higher than that of MAcD or TAcD.

The peak of daily methane yield occurred earlier under thermophilic conditions, indicating that higher temperatures greatly improve the hydrolysis rate, rapidly breaking down the cell walls of organic matter, and accelerating subsequent processes^27^. The cumulative methane yield of TPAcD increased by 50.3% compared to MAcD, and by 32.7% compared to TAcD. These experimental results demonstrate that TPAcD is beneficial for the anaerobic co-digestion of sewage sludge and food waste, with significant improvements in both methane production rate and cumulative methane yield.

### Variations of VFA and pH

**Figure 4:**
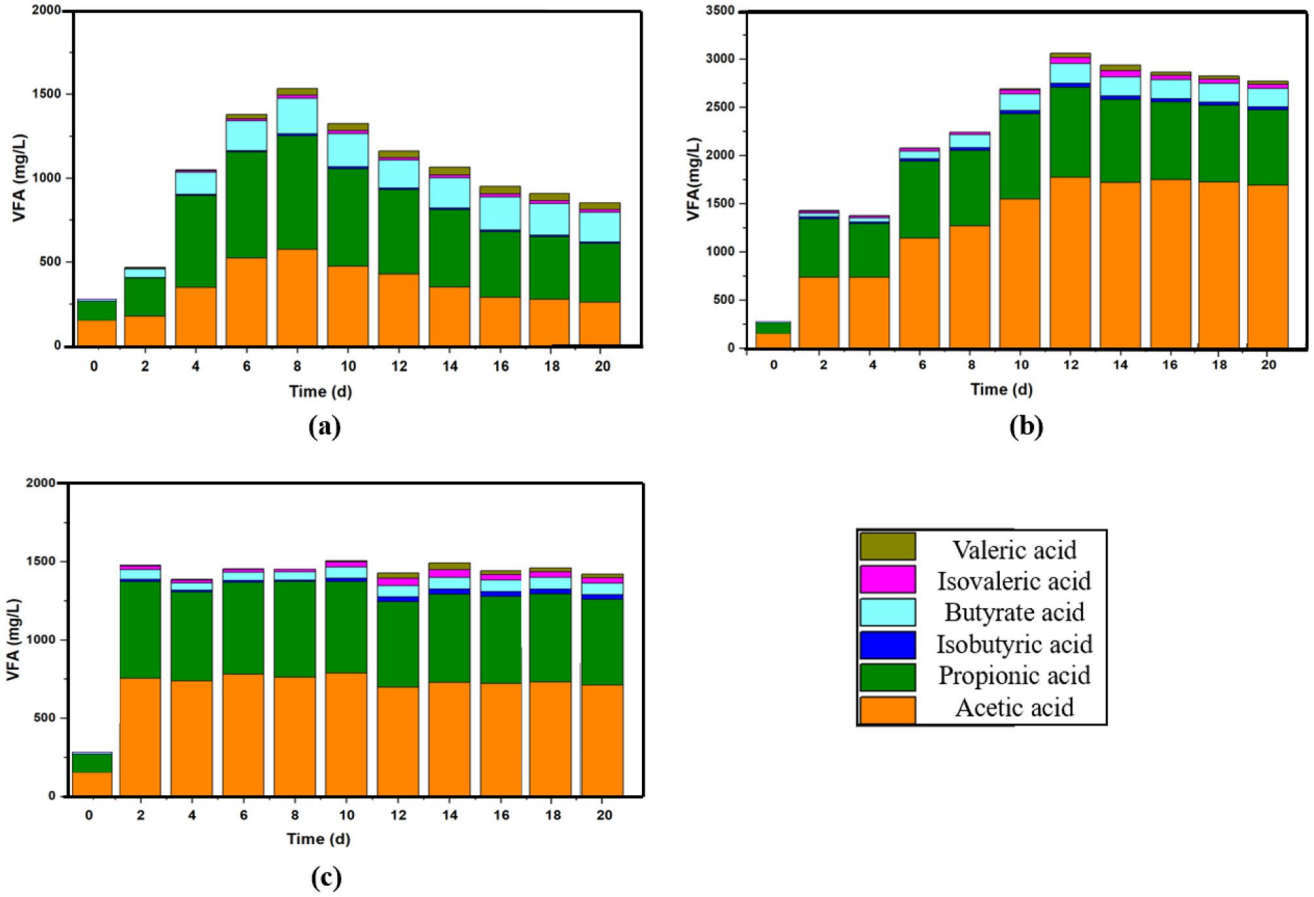
Variations of VFA of (**a**) MAcD; (**b**) TAcD; (**c**) TPAcD.

During the anaerobic digestion process, large organic molecules such as proteins, carbohydrates, fats, oils, and grease are broken down into smaller, soluble organic molecules such as sugars, amino acids, and fatty acids in the hydrolysis stage. These soluble organic molecules are then converted into VFA in the acidogenesis stage. The VFA is further degraded into hydrogen and acetic acid in the acetogenesis stage. Finally, methane is produced from the acetic acid and hydrogen in the methanogenesis stage^28^. VFA plays a crucial role in altering the anaerobic digestion process and achieving stable methane production, as well as synchronizing the series of biochemical reactions^29^. Six main types of VFA (acetic acid, propionic acid, isobutyric acid, butyrate acid, isovaleric acid, and valeric acid) were analyzed to determine the progress of the process. Figure 4 shows the changes in VFA levels during the MAcD, TAcD, and TPAcD processes.

As shown in Fig. 4a, the VFA concentration of MAcD was relatively low, with acetic acid and propionic acid being the dominant VFAs, accounting for over 80%. The VFA concentration increased in the initial stage, peaked on the 8th day, and gradually decreased in the following days. In contrast, the VFA concentration of TAcD remained relatively high, as illustrated in Fig. 4b. However, the daily methane yield of TAcD was very low after the 11th day. This can be attributed to a significant increase in acetic acid concentration, while the concentrations of other acids remained almost unchanged. This suggests that while the acetogenesis stage was not inhibited, the methanogenesis stage was severely inhibited. At thermophilic temperatures, acidifying bacteria convert products to VFA at a faster rate than methanogenesis can consume them, leading to VFA accumulation and a rapid decrease in pH, which inhibits methanogenic bacteria^30^. Figure 4c displays the VFA variation of TPAcD. Under thermophilic conditions (55 °C), the VFA concentration rapidly reached a moderate level and remained stable under mesophilic conditions (35 °C). The use of NaOH as a buffering agent prevented the pH from dropping to levels that would inhibit the activity of methanogenic bacteria, resulting in a more sustainable and steady methane production.

**Figure 5:**
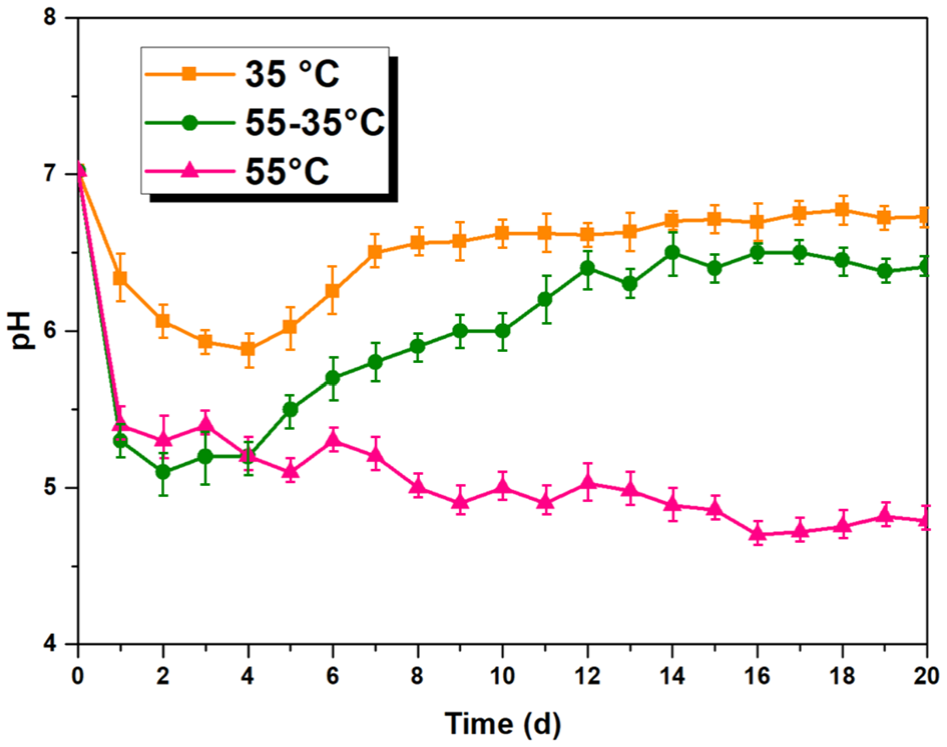
Variation of pH.

The variations of pH and VFA mutually corroborate each other. Figure 5 illustrates the pH fluctuations of three types of AcD. In the MAcD (35 °C) process, there was a small initial increase in VFA production, which caused a slight decrease in pH. The following increase in pH was due to the consumption of VFA by methanogenesis stage and ammonia production, which adsorbed the protons (H^+^) in the AD process^31^. This resulted in a gradual increase in pH over the following days. By the 8th day, the pH stabilized, while the VFA concentration and daily methane yield decreased slowly. In the TAcD (55 °C) process, a large amount of acid is produced under thermophilic conditions. However, the rate of methane production is not able to keep up with the rate of acid production, leading to a continuous decrease in pH that falls outside the suitable range for the metabolism of methanogenic bacteria^26^. In the TPAcD (55–35 °C) process, the early-stage pH fluctuations were similar to those of TAcD. However, in the later stage under mesophilic conditions, the pH gradually increased and maintained a suitable level close to that of MAcD.

From the variations of VFA and pH, it’s proved that TPAcD has both a higher hydrolysis and acidification rate in the early stage under thermophilic condition and a suitable pH range in the later stage under mesophilic condition, so the methane yield of TPAcD is better than that of single phase MAcD or TAcD.

### Variation of ammonia nitrogen

In anaerobic digestion, nitrogen is present in two forms: organic and inorganic. Organic nitrogen is found in the form of proteins and amino acids. During the hydrolysis process, organic nitrogen breaks down into ammonia nitrogen (NH^4+^–N)^32^. It is important to analyze the levels of ammonia nitrogen in AD for several reasons. Firstly, ammonia nitrogen can neutralize VFA produced. Secondly, it is a crucial nutrient for the growth of AD microorganisms and the synthesis of cells. Thridly, excessive levels of ammonia nitrogen can be toxic to methanogens, inhibiting their metabolism and ultimately affecting methane production^33^.

**Figure 6:**
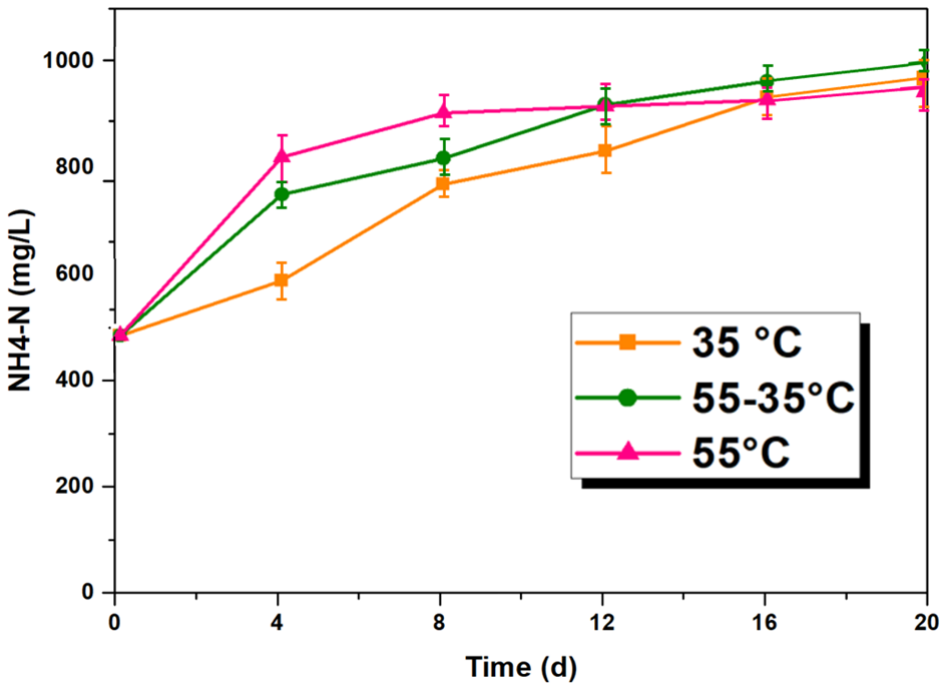
Variation of ammonia nitrogen.

Figure 6 illustrates the changes in ammonia nitrogen levels during the AD process. The concentration of ammonia nitrogen in MAcD (35 °C) gradually increased over time. Attributed to the rapid hydrolysis and acidification that occurs at thermophilic temperatures (55 °C), the ammonia nitrogen levels in TAcD increased rapidly in the initial days. This was accompanied by the production of a large amount of VFA, which were then neutralized by the ammonia nitrogen. As a result, there was no significant fluctuation in the ammonia nitrogen levels in the later stages of the process. The ammonia nitrogen generation rate in TPAcD (55–35 °C) under thermophilic conditions was similar to that of TAcD, but decreased after switching to mesophilic conditions. These findings suggest that the AD process leads to an increase in ammonia nitrogen levels, and that excessively high or low generation rates may hinder methane production. A lower generation rate can result in a lack of nutrients for microbial growth, which can affect the stability of the process. On the other hand, a higher generation rate can cause the ammonia nitrogen levels to exceed the threshold, leading to a significant decrease in microbial growth^34^.

### Kinetic parameters of methane production

The experimental data were fitted to the Gompertz model to evaluate methane production in MAcD, TAcD, and TPAcD. As shown in Table 2, the high R^2^ value indicates reliable prediction accuracy. The thermophilic conditions greatly enhanced the maximum methane production rate and reduced the lag time. These results suggest that thermophilic temperatures accelerate the AcD process and optimize the kinetic parameters. Among the three AcDs, TPAcD showed the highest potential and rate for methane production. While TAcD had a short lag time, the rapid and large accumulation of VFA affected its maximum methane production potential. Overall, TPAcD demonstrated better methane production performance compared to MAcD and TAcD.

**Table 2:** Kinetic parameters of methane production in MAcD, TAcD and TPAcD.

Parameters	MAcD	TAcD	TPAcD
V_m_/(mL g^−1^ VS)	204.1	224.7	298.6
S/(mL g^−1^ d^−1^)	22.2203	38.4796	40.7449
D/d	1.4884	0.6279	0.8256
R^2^	0.9968	0.9992	0.9987

### Comparison of thermal pretreatment and TPAcD

Thermal pretreatment and TPAcD have some similarities, as they both involve high temperatures. However, thermal pretreatment is conducted in an aerobic environment and at higher temperatures than thermophilic temperatures. While thermal pretreatment does increase the soluble chemical oxygen demand (SCOD) of substrates, the resulting increase in methane production during AD is not significant enough to match the substantial increase in SCOD^35^. For example, after thermal pretreatment for 24 h at 60, 70, 80, and 90 °C, SCOD/TCOD increased from 4.5% to 29.6%, 30.3%, 34.8%, and 41.1%, respectively, while biogas production only increased only by 11.3%, 9.3%, 12.3%, and 8.8%, respectively^36^. Additionally, high temperature pretreatment (> 100 °C) requires high quality heat and results in higher operating costs^37^. In comparison, TPAcD has a better performance in enhancing methane production (increased by 50.3% and 32.7% compared to MAcD and TAcD, respectively) with acceptable operating costs. Furthermore, TPAcD reduces the filtration time of digestate dewatering and improves biodegradation compared to low temperature pretreatment, due to the presence of active hydrolytic enzymes^37^.

### Electricity generation and avoided GHG emissions

The potential for electricity generation (EG) and avoided greenhouse gas (GHG) emissions for the MAcD, TAcD, and TPAcD were calculated and are shown in Table 3. The TPAcD system had a higher electricity generation compared to the single-phase systems due to its higher methane production. Other studies have also reported higher energy recovery from two-phase systems compared to single-phase systems^38,39^. In terms of emissions avoided, the electricity generated from the TPAcD system could mitigate 48.51 kg CO_2_eq/t. In the context of “carbon neutrality”, the significant advantage of the energy produced from TPAcD using sewage sludge and food waste is its ability to replace other energy sources and reduce associated GHG emissions.

**Table 3:** The potential of electricity generation (EG) and avoided GHG emissions of MAcD, TAcD and TPAcD.

Parameters	MAcD	TAcD	TPAcD
EG (kWh/t)	120.25	131.96	175.26
A_GHG_ (kg CO_2_eq/t)	33.29	36.53	48.51

## Conclusion

This paper investigates the anaerobic co-digestion of sewage sludge and food waste. After exploring the appropriate ratio to achieve the optimal C/N of the mixture, a comparison of MAcD, TAcD, and TPAcD was conducted at the optimal C/N. The experimental results showed that the optimal mixture ratio of sewage sludge and food waste was 40:60, resulting in an optimal C/N of 15.5. At this mixture ratio, the methane yield of TPAcD increased by 50.3% and 32.7% compared to MAcD and TAcD, respectively. Based on the analysis of process parameters and kinetic parameters, TPAcD was found to be beneficial for the anaerobic digestion of sewage sludge and food waste, with significant improvements in methane production rate, total methane yield, and system stability. Additionally, TPAcD has the potential to reduce GHG emissions and contribute to achieving “carbon neutrality”.

## Data Availability

The datasets used and/or analysed during the current study available from the corresponding author on reasonable request.
